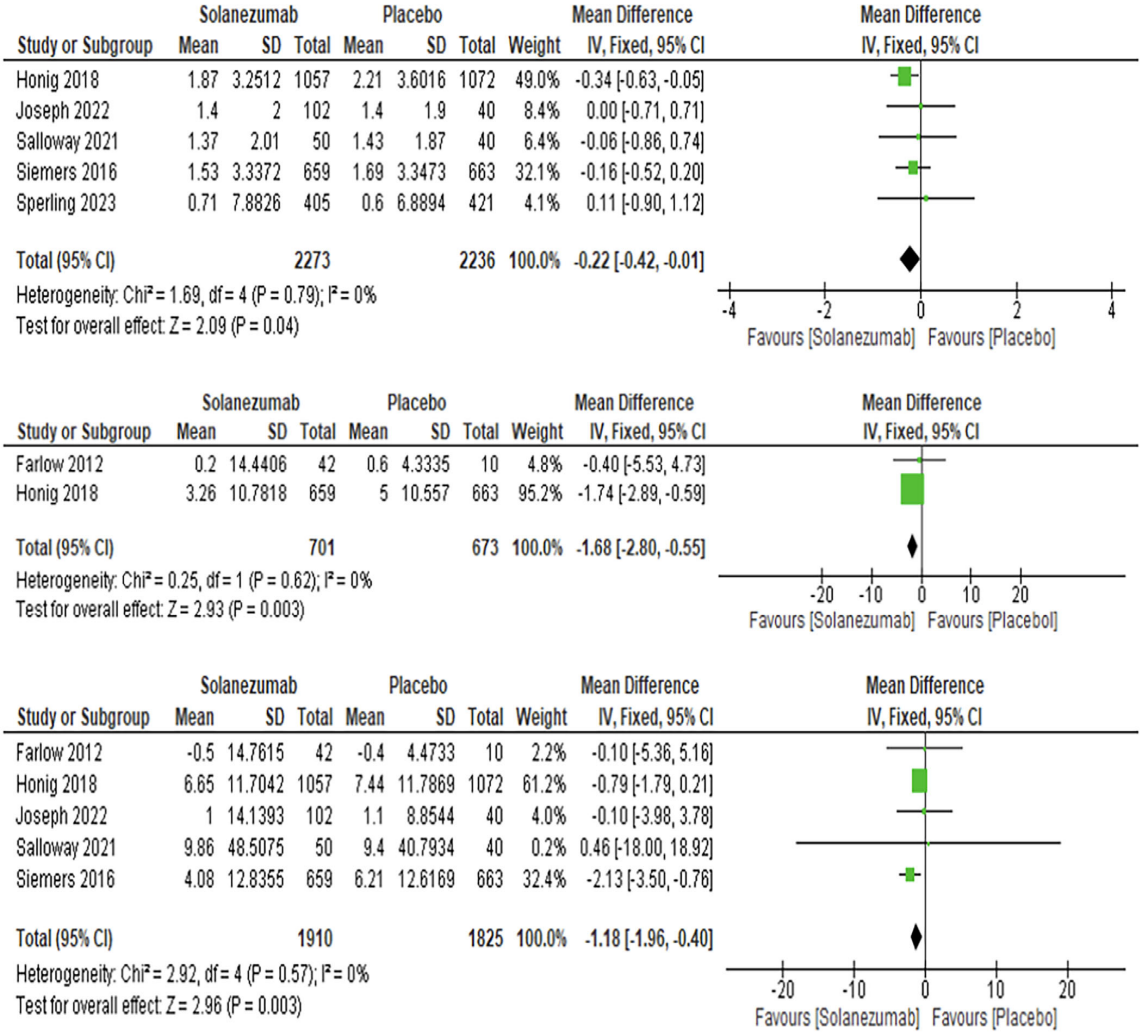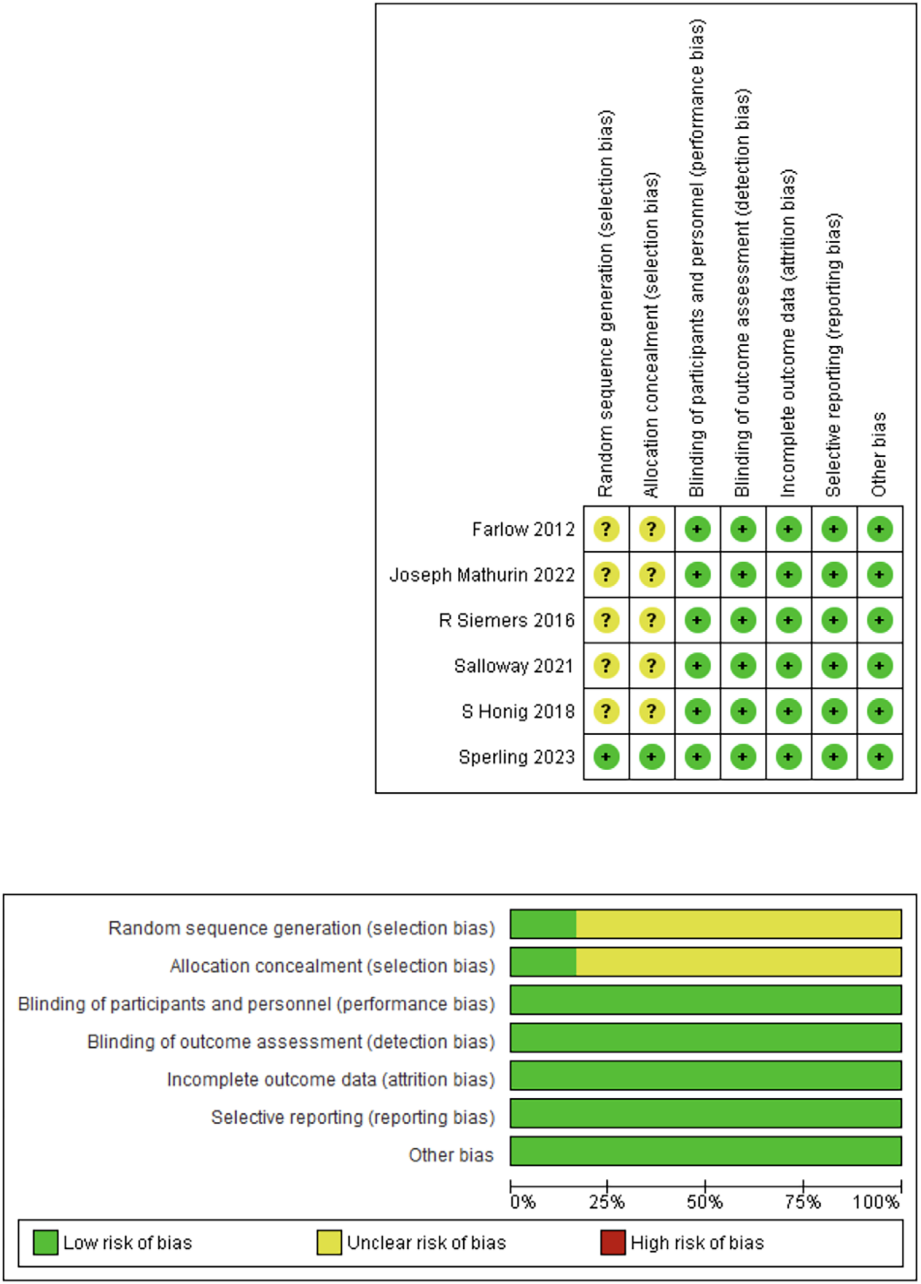# Solanezumab Treatment for Alzheimer disease : Meta analysis for Non EXPEDITION Trials

**DOI:** 10.1002/alz.084029

**Published:** 2025-01-09

**Authors:** AMEER FADHIL ABBAS AL WSSAWI, Ali Saad Al‐Shammary, Sahar Imtiaz, Maham Rana, Mahmoud Gamal, Mahmoud Moustafa Ahmed Amin, Rawan Mohammad Ibrahim Nawas

**Affiliations:** ^1^ Al‐Qadisiyah university / College Of Medicine, بابل Iraq; ^2^ Baghdad medical city, Baghdad Iraq; ^3^ Dow Medical College, Dow Pakistan; ^4^ Faculty of medicine ‐ Fayoum University, Fayoum Egypt; ^5^ Quds university, Quds Palestine

## Abstract

**Background:**

Solanezumab, a promising treatment for Alzheimer’s disease, has captured the attention of the medical community. This monoclonal antibody is designed to target and clear beta‐amyloid plaques, a hallmark feature of Alzheimer’s, from the brain. While initial clinical trials showed mixed results, ongoing research is exploring its potential to slow cognitive decline and improve the lives of those affected by this devastating neurodegenerative condition.

**Method:**

A systematic review and meta analysis was conducted and 6 studies out of 1202 were included. The total number of the patients were 4956 And all the studies were Randomised clinical trials. 5 RCTs included, outcome of (ADAS‐Cog14, ADAS‐Cog11, MMSE, CDR‐SB Score), intervention number in each Trial (n1 = 578, n2 = 102, n3 = 50, n4 = 1057, n5 = 659), Analysis was done by Review manager program version 5.0 and I2 of less than 0.05 was considered significant.

**Result:**

Improvements in cognitive subscale of the Alzheimer’s Disease Assessment Scale (ADAS‐cog14) were observed by 5 studies (Farlow, Honig, Joseph, Salloway, Siemers). The overall pooled results showed that Solanezumab is associated with a significant reduction in ADAS‐ Cog 14 scores as compared to placebo. (MD ‐1.18,95% CI(‐1.96,‐0.40); p = 0.003, I2 = 0%). ADAS‐Cog11: Improvements in cognitive subscale of the Alzheimer’s Disease Assessment Scale (ADAS‐cog11) were observed by 2 studies(Farlow, Honig,). Pooled analysis showed that Solanezumab is associated with a significant reduction in ADAS‐ Cog 11 scores as compared to placebo. (MD ‐1.68,95% CI(‐2.80,‐0.55); p = 0.003, I2 = 0%) CDR‐SB :Clinical Dementia Rating Sum of Boxes (CDR‐SB) is reported by 5 studies (Honig, Joseph, Salloway, Siemers, Sperling). The overall pooled results showed that Solanezumab led to substantial improvement in CDR‐SB as compared to placebo. (MD ‐0.22,95% CI(‐0.42,‐0.01); p = 0.04, I2 = 0%).

**Conclusion:**

Solanezumab’s potential as an Alzheimer’s disease treatment has spurred considerable scientific interest and debate. This monoclonal antibody specifically targets beta‐amyloid plaques, a prominent feature in Alzheimer’s brains. While initial clinical trials showed disappointing results in terms of halting cognitive decline, some argue that the drug may still have promise when administered at earlier stages of the disease or in combination with other therapies.